# Effect of Cationic (Na^+^) and Anionic (F^−^) Co-Doping on the Structural and Electrochemical Properties of LiNi_1/3_Mn_1/3_Co_1/3_O_2_ Cathode Material for Lithium-Ion Batteries

**DOI:** 10.3390/ijms23126755

**Published:** 2022-06-17

**Authors:** Hua Wang, Ahmed M. Hashem, Ashraf E. Abdel-Ghany, Somia M. Abbas, Rasha S. El-Tawil, Tianyi Li, Xintong Li, Hazim El-Mounayri, Andres Tovar, Likun Zhu, Alain Mauger, Christian M. Julien

**Affiliations:** 1 Department of Mechanical and Energy Engineering, Indiana University-Purdue University Indianapolis, Indianapolis, IN 46202, USA; wanghua@iu.edu (H.W.); xl80@iu.edu (X.L.); helmouna@iupui.edu (H.E.-M.); tovara@iupui.edu (A.T.); likzhu@iupui.edu (L.Z.); 2 Inorganic Chemistry Department, National Research Centre, 33 El Bohouth St., (Former El Tahrir St.), Dokki, Giza 12622, Egypt; ahmedh242@yahoo.com (A.M.H.); achraf_28@yahoo.com (A.E.A.-G.); somiamohamed119@yahoo.com (S.M.A.); r2samir@yahoo.com (R.S.E.-T.); 3 Advanced Photon Source, Argonne National Laboratory, Lemont, IL 60439, USA; tianyi.li@anl.gov; 4 Institut de Minéralogie, de Physique des Matériaux et Cosmologie (IMPMC), Sorbonne Université, UMR-CNRS 7590, 4 Place Jussieu, 75752 Paris, France; alain.mauger@sorbonne-universite.fr

**Keywords:** LiNi_1/3_Mn_1/3_Co_1/3_O_2_, Na/F co-doping, layered oxide, cathode material, long-life cycling, lithium-ion batteries

## Abstract

Elemental doping for substituting lithium or oxygen sites has become a simple and effective technique to improve the electrochemical performance of layered cathode materials. Compared with single-element doping, this work presents an unprecedented contribution to the study of the effect of Na^+^/F^−^ co-doping on the structure and electrochemical performance of LiNi_1/3_Mn_1/3_Co_1/3_O_2_. The co-doped Li_1-z_Na_z_Ni_1/3_Mn_1/3_Co_1/3_O_2-z_F_z_ (z = 0.025) and pristine LiNi_1/3_Co_1/3_Mn_1/3_O_2_ materials were synthesized via the sol–gel method using EDTA as a chelating agent. Structural analyses, carried out by X-ray diffraction, Raman spectroscopy, and X-ray photoelectron spectroscopy, revealed that the Na^+^ and F^−^ dopants were successfully incorporated into the Li and O sites, respectively. The co-doping resulted in larger Li-slab spacing, a lower degree of cation mixing, and the stabilization of the surface structure, which substantially enhanced the cycling stability and rate capability of the cathode material. The Na/F co-doped LiNi_1/3_Mn_1/3_Co_1/3_O_2_ electrode delivered an initial specific capacity of 142 mAh g^−1^ at a 1C rate (178 mAh g^−1^ at 0.1C), and it maintained 50% of its initial capacity after 1000 charge–discharge cycles at a 1C rate.

## 1. Introduction

Lithium-ion batteries (LIBs) have been widely used as the power source for portable electronics and electric vehicles due to their high energy and power densities, long life cycle, and low self-discharge [1]. The cathode material plays a critical role in determining the electrochemical performance of LIBs [2,3]. In recent years, LiNi_1/3_Mn_1/3_Co_1/3_O_2_ (NMC333), first proposed by Ohzuku [4] and Shaju [5], has been widely used in LIBs for electric vehicles due to its high capacity. However, some disadvantages still remain for NMC333, including poor rate capability, significant initial irreversible capacity loss, and low thermal stability at high-cutoff voltage [6]. To address these problems, many modification methods have been studied, such as surface modification [7] and ion doping [8,9], which can minimize the cationic (Li^+^/Ni^2+^) mixing, enhance the electrochemical efficiency of NMC333, and stabilize the intercalated host structure. Ion doping can be achieved by introducing substitution ions into the transition metal (TM) sites: the Li site or O site of NMC333. For instance, cationic substitution or doping on the TM sites has been performed using ions such as Al^3+^ [10], Cr^3+^ [11], Mg^2+^ [8,12], Zn^2+^ [9], Zr^4+^ [13], Ti^4+^ [14], etc. The replacement of Li by another monovalent cation with a larger ionic radius, such as Na^+^ [15,16,17,18] and K^+^ [19], has also been investigated. Anionic doping has been performed by substituting oxygen with F^−^ [20,21,22,23], Cl^−^ [24], and (PO_4_)^3-^ polyanions [25].

Among the doping elements, Na^+^ and F^−^ are the commonly investigated cation and anion, respectively. Recent studies have demonstrated that elemental substitution with Na and F can enhance the structural stability and improve the electrochemical performance of NMC333. For instance, first-principles calculations of the 3D and 2D potential maps demonstrated that Na doping can reduce the potential well and increase the removal rate of Li^+^ ions [26]. Li et al. [17] reported that Li_1-x_Na_x_Ni_1/3_Mn_1/3_Co_1/3_O_2_ improved the electrochemical performance compared with undoped material. Gong et al. [15] reported that Li_0.95_Na_0.05_Ni_1/3_Mn_1/3_Co_1/3_O_2_ has a much higher capacity than the undoped material. In addition, the Na-doped material shows the capability of self-repairing. Substituting Li with Na can not only significantly preserve the layered structure of NMC333, but also promote Li-ion diffusion by enlarged Li-layer spacing due to the larger ionic radius of Na and reduced Li^+^/Ni^2+^ cation mixing, which thereby improves the cycling durability and rate capability [27]. He et al. [21] reported that anion-doped NMC333 using fluorine (LiNi_1/3_Mn_1/3_Co_1/3_O_2-_*_z_*F*_z_*) has a lower initial discharge capacity but better cycling stability and rate capability than the undoped one. They proposed that F doping can stabilize the crystal structure by protecting the surface of the cathode material from HF attack through electrolyte dissociation and oxygen loss in the lattice, as well as suppress the phase transition from the layered to spinel phase. F doping can also reduce the charge-transfer resistance and enhance Li-ion diffusion, which are beneficial for improving the electrochemical performances of layered cathode materials, and especially the rate capability and reversible capacity [28,29,30].

Recently, Na/F co-doping is becoming a promising strategy to maintain the advantages of both cation and anion doping to improve the structural stability and electrochemical performance of Li-rich, Ni-rich, or Li, Mn-rich layered-structure cathode materials [27,31,32,33]. To the best of our knowledge, Na^+^/F^−^ co-doping has not been examined on NMC333. The objective of this paper is to elucidate the effect of Na^+^/F^−^ co-doping on the electrochemical behavior of a layered NMC333 cathode material. This study aims at investigating the structure, physical chemistry, and electrochemical properties of the Li_1-z_Na_z_Ni_1/3_Mn_1/3_Co_1/3_O_2-z_F_z_ (*z* = 0.025) (Na/F-NMC333) material synthesized via the sol–gel method using EDTA as a chelating agent. The as-prepared Na/F-NMC333 cathode material showed excellent capacity retention, rate capability, and cycling stability when tested over 1000 cycles.

## 2. Results and Discussion

### 2.1. Structure and Morphology

**Figure 1:**
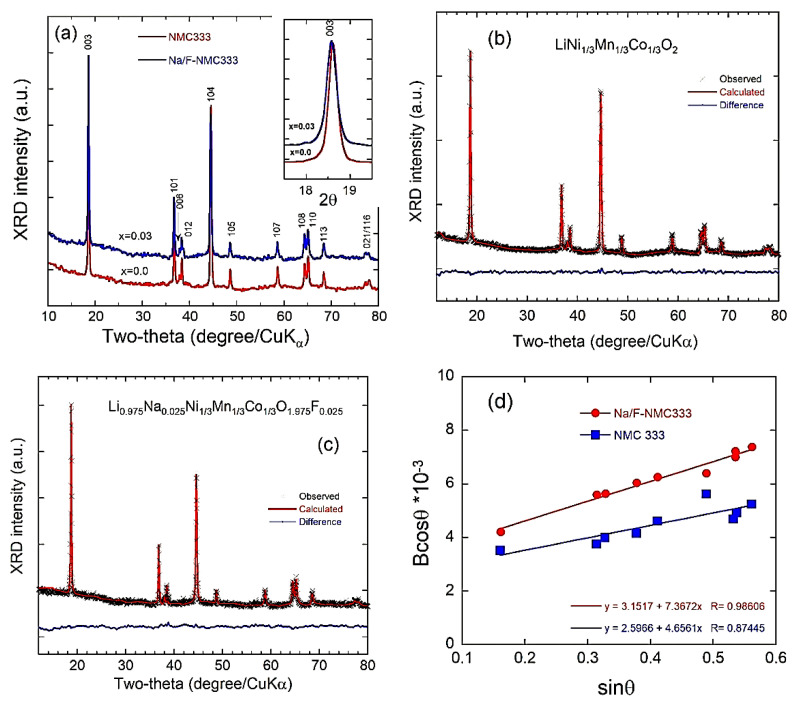
(**a**) XRD patterns of NMC333 and Na/F-NMC333 obtained from X-ray radiation with a photon wavelength of 1.54056 Å. Rietveld refinements of X-ray diffractograms for: (**b**) pristine NMC333 and (**c**) Na/F-NMC333. (**d**) Analysis of the microstrain from the broadening of Bragg reflections according to Equation (1).

Figure 1a shows the X-ray diffraction (XRD) patterns of the as-synthesized NMC333 and Na/F-NMC333. All the peaks in both samples adopt a single-phase layered structure, with the *R*$\bar{3}$*m* space group of the hexagonal α-NaFeO_2_ (standard card JCPDS 82-1495) [34]. This well-developed and good crystalline–cation-ordering layered structure can be confirmed from the distinct splitting of the (108)/(110) and (006)/(102) doublets, high-intensity ratio between the (003) and (104) peaks, and high *c/a* lattice ratio [35]. Here, cation ordering was identified from two characters: c*/a* > 4.94 and a high peak ratio (*I*_003_/*I*_104_), which are good indicators for a lower amount of undesirable cation mixing and a better hexagonal structure [35]. These two factors are higher in the co-doped sample (Na/F-NMC333), as is shown in Table 1. All these parameters are beneficial to the electrochemical properties of the host lattice. A careful analysis of the XRD spectra indicates that the (003) peak of the Na/F-NMC333 sample moves slightly toward the lower-angle region, as is shown in the inset of Figure 1a. This slight change confirms that the Na^+^ ions were introduced into the lithium layer in the lattice, which may enlarge the Li-slab space substantially. Due to its larger ionic radius (*r*_Na_ = 1.02 Å), Na^+^ cannot substitute for Ni^2+^, Co^3+^, and Mn^4+^, which have much smaller radii (0.69, 0.545, and 0.53 Å, respectively).

Further analyses of the XRD patterns of the prepared samples were performed by Rietveld refinement. It is assumed that, in the *R*$\bar{3}$*m* structure: the 3*a* sites are occupied by Li^+^, Na^+^, and a small quantity of Ni^2+^ cations; the transition-metal ions Ni, Mn, and Co are located in the 3*b* site; and O^2−^ and F^−^ anions are located in the 6*c* site [16,36]. Therefore, the total occupation of the 3*a* and 3*b* sites should be equal to 1, while the total occupancy of 6c sites is (O)_6c_ + (F)_6c_ = 2, and the structural formulae of the Na/F-NMC333 sample is assumed to be [Li_1-x-y_Na_x_Ni_y_]_3a_[Ni_1/3-y_Mn_1/3_Co_1/3_]_3b_[O_2-x_F_x_]_6c_. The refined XRD spectra are displayed in Figure 1b,c for NMC333 and Na/F–NMC333, respectively, where cross marks are the experimental data and solid lines (in red) are the calculated spectra. The difference between the calculated and experimental diffractograms (blue curves) shows the quality of the fit, which validates the structural model.

The lattice parameters obtained from the Rietveld refinement (Table 1) are in good agreement with the values previously reported [16,35]. The values of both the *a*- and *c*-axis parameters are slightly enlarged (i.e., 0.2 and 0.1%, respectively) upon co-doping by Na and F. The successful incorporation of Na into the Li site reduces the Ni/Li cation mixing. The percentage of Ni^2+^ ions in the Li layer (1.28%) for Na/F-NMC333 is lower than that observed for pristine NMC333 (2.08%), which attests to the beneficial effect of the incorporation of Na^+^ ions into the Li site. The cationic-mixing mitigation is also emphasized by the higher *c*/*a* ratio and the enhanced peak intensity ratio (*I*_003_/*I*_104_) for the co-doped Na/F-NMC333 sample. The thicknesses of the TM slab (*S*_(MO2)_) and that of the interslab (*I*_(LiO2)_) were calculated to further investigate the structural properties of NMC333 and Na/Fe-NMC333 in more detail. We notice that *S*_(MO2)_ is reduced by 4.6%, whereas *I*_(LiO2)_ is enlarged by 7.2% compared with those of the pristine NMC333 sample. This is more evidence for the replacement of Na^+^ ions (*r*_Na+_ = 1.02 Å) with Li (*r*_Li+_ = 0.76 Å), rather than their insertion into the TM layer and the substitution of fluorine (with ionic radius: *r*_F−_ = 1.33 Å) for oxygen (*r*_O2−_ = 1.40 Å) in the 6*c* Wyckoff site. Xiang et al. [31] claim that the substitution of F^−^ for O^2−^ explains the increase in the lattice parameters *a* and *c*, which is implied by this substitution (even though *r*_F− <_ *r*_O2−_). The increasing *I*_(LiO2)_ (+7.2%) could lead to the rapid diffusion of lithium ions and improve the electrochemical performance because the larger *I*_(LiO2)_ enhances the diffusion coefficient of Li^+^ ions [9]. Moreover, the decrease in *S*_(MO2)_ caused by the substitution of F in oxygen sites improves the structural stability, which, in turn, mitigates the TM dissolution.

Further information on the structural properties can be obtained from the broadening of the diffraction peaks that is considered an indicator not only of the crystallinity of NMC333 and Na/F-NMC333 powders, but also of the homogeneous distribution of cations within the structure. The microstrain (*ε*) of the NMC333 and Na/F-NMC333 particles was determined using the Williamson–Hall equation [37]:

*B*_hkl_ cos *θ*_hkl_ = (*K*λ/*L*_c_) + 4*ε* sin *θ*_hkl_ (1)

where *B*_hkl_ is the line broadening of a Bragg reflection (*hkl*); *K* = 0.9 is the shape factor for a spherical particle; *L*_c_ is the crystallite size; λ is the X-ray wavelength. The average crystallite size of the as-prepared samples calculated using the Debye–Scherrer formula from the full-width at half-maximum of the seven main reflections are 42 and 34 nm for NMC333 and Na/F-NMC333, respectively. The microstrain is estimated from the slope of the plot ((*B*_hkl_ cos *θ*_hkl_) vs. (4 sin *θ*_hkl_)), and the intersection with the vertical axis provides the crystallite size. The *B*_hkl_ value used here is the instrumental corrected one. As shown in Figure 1d, *ε* = 1.16 × 10^−3^ and 1.84 × 10^−3^ rd for NMC333 and Na/F-NMC333, respectively. This is due to the fact that the substitution of Li with Na enlarges the interslab spacing distance of LiO_2_ (*I*_LiO2_) for Na/F-NMC333, and our results are in agreement with the prior work [15,16,17,18]. It should be noted that the lattice strain is not due to any “flux”. It is the consequence of the incorporation of Na that does not have the same ionic radius as Li [15,16,17,18], as well as the effect of the charge compensation related to the substitution of O^2−^ with F^−^ [31]. Moreover, according to Vegard’s law, the Na concentration can be up to about 5%. The concentration of Na that we chose (2.5%) is below the limit of solubility of Na in this material.

**Figure 2:**
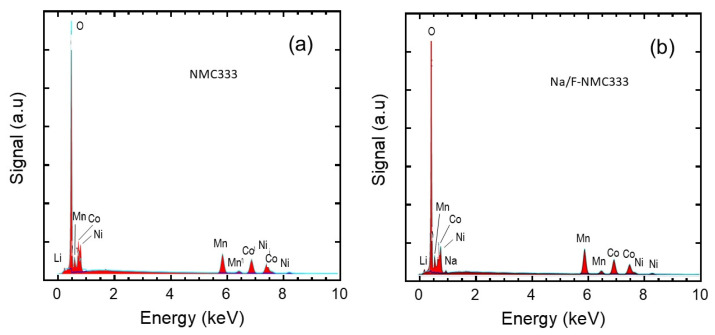
SEM–EDS spectra of: (**a**) pristine LiNi_1/3_Mn_1/3_Co_1/3_O_2_ and (**b**) Na/F co-doped Li_1-z_Na_z_Ni_1/3_Mn_1/3_Co_1/3_O_2-z_F_z_ (z = 0.025).

**Figure 3:**
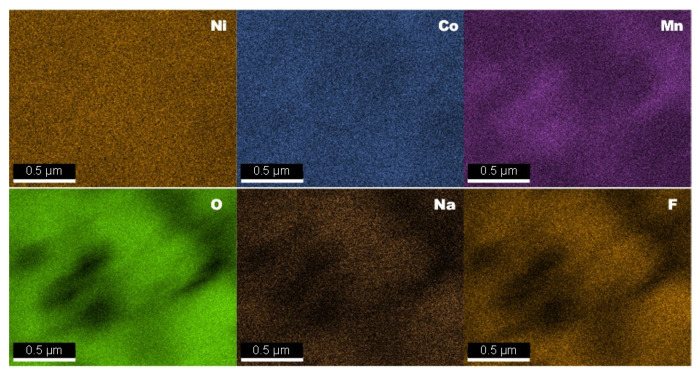
Elemental mapping of Ni, Co, Mn, O, Na, and F for the Na/F co-doped NMC333 sample from SEM–EDS.

The elemental compositions of the pristine NMC333 and co-doped Na/F-NMC333 samples were determined using an energy-dispersive X-ray spectrometer attached to the SEM apparatus (SEM–EDS). The SEM–ESD analyses are depicted in Figure 2. Due to the very low content of fluorine and the vicinity of its K_α_ peak at 0.677 keV, the signal is mingled with the stronger L_α_ peak of Mn at 0.637 keV. The EDS spectra indicate that the compositions of the samples are in agreement with the nominal formula (see results in Table 2). In the co-doped Na/F-NMC333 sample, the sodium and fluorine molar fractions are 0.028 and 0.026, respectively, which match well with the nominal composition (*z* = 0.025). Note that the co-doped material contains less Ni^2+^ cations than the pristine sample. Figure 3 displays the elemental mapping of the Na/F-NMC333 sample. Ni, Co, and Mn are uniformly distributed in both samples. The Na and F elements are almost homogeneously distributed, although some Na- and F-rich regions can be observed. This feature results from the mixing method, which is very difficult to handle for a uniform atomic spatial distribution [38].

**Figure 4:**
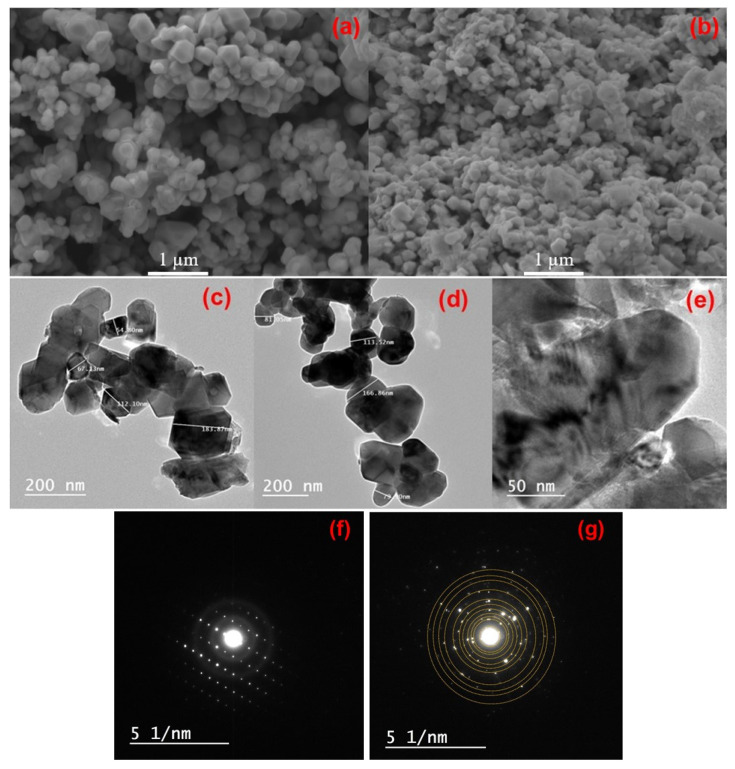
(**a**,**b**) SEM images, (**c**–**e**) TEM images, and (**f**,**g**) SAED patterns of pristine (**a**,**c**,**f**) NMC333 and (**b**,**d**,**e**,**g**) Na/F-NMC333 powders.

The morphology of the as-prepared samples was investigated through scanning and transmission electron microscopies. The SEM and TEM images of the NMC333 and Na/F-NMC333 samples shown in Figure 4a–d, respectively, display the typical morphology of nanostructured particles (submicron in size) synthesized by the sol–gel method. This technique, using EDTA as a chelating agent, can effectively control a homogeneous particle size distribution. The SEM images reveal the regular polygon shape of the nanoparticles. At the same time, the introduction of dopants into the NMC333 network maintains a uniform distribution of the particles, with a small decrease in the average nanoparticle size. The faceted nanoparticles remain unaltered with co-doping. The TEM images for NMC333 and Na/F-NMC333, displayed in Figure 4c,d, respectively, confirm the above observations. The TEM patterns (200 nm scale) do not show a significant difference in the particle shape upon co-doping in the NCM333 layered framework. The nanoparticles appear well-faceted, and the average particle sizes were found to be 135 nm for pristine NMC333, and 121 nm for co-doped Na/F-NMC333. Figure 4e displays the HRTEM image of Na/F-NMC333 (50 nm scale), in which no LiF or NaF coating was detected at a very low F loading (*z* = 0.025). This lack of surface coating is consistent with previous reports [39,40]. In addition, several papers mention that F substitution reduces LiF formation in the CEI layer, which enhances the Li-ion transfer at the electrolyte/electrode interface. In contrast, the existence of a LiF surface layer substantially alters the ionic transfer, which is due to its poor Li-ion conductivity [39].

The selected-area electron diffraction (SAED) patterns of the NMC-333 and Na/F-NMC333 sample powders are shown in Figure 4f,g. The SAED consists mainly of one type of reflection, which reveals that the individual particles have the single-crystalline structure of the rhombohedral (*R*$\bar{3}$*m*) phase. In addition, weak reflections are observed for the Na/F-NMC333 sample, which indicates that a region of polycrystalline domain was observed.

**Figure 5:**
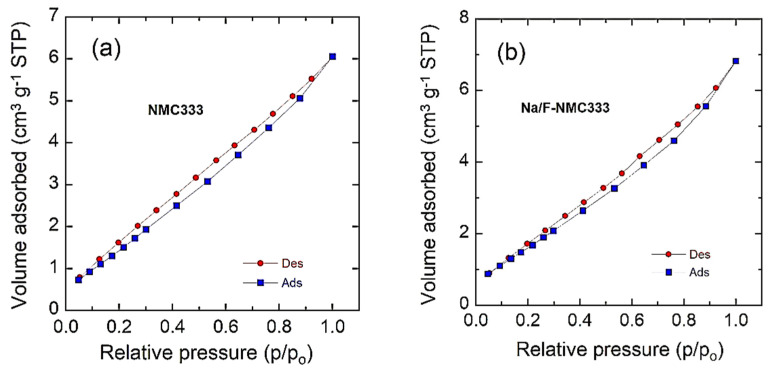
BET experiments showing N_2_ adsorption–desorption isotherms of: (**a**) pristine NMC333 and (**b**) Na/F-NMC333 samples.

The specific surface area (SSA) of an electrochemically active material is an important parameter for the determination of the exchange-current at the electrolyte–electrode interface and the kinetics of Li^+^ ions in the electrode. The SSAs of the pristine and co-doped NMC333 powders were measured by the nitrogen adsorption method. The results are shown in Figure 5. The BET specific surface area (*S*_BET_) of pristine NMC333 is 6.7 m^2^ g^−1^, which slightly increases to 7.1 m^2^ g^−1^ with Na/F co-doping. The isotherm curves of both samples display hysteresis loops, which indicate the hierarchical nanoporous structure of the powders. The nanopores (~3 nm in size) calculated using the Barrett–Joyner–Halenda (BJH) model correspond to the interconnecting voids that exist between randomly packed nanoparticles. All isotherms increase with increasing *p*/*p*_0_ and form a H3-type hysteresis loop up to *p/p*_0_ ≈ 0.98, according to the IUPAC classification [41]. The average diameter (in nm) of particles can be estimated from BET measurements using the relation [42]:

(2) $$L_{BET}=\frac{6000}{S_{BET\;}d}$$

where *d* is the gravimetric density (*d* = 4.55 g cm^−3^ for NMC333 material). The values of *L*_BET_ match well with the particle sizes evaluated from the TEM patterns (Table 3). It is also shown that the Na/F-NMC333 powder has an open porosity, and this ensures a better wettability relating to penetration through the electrolyte, and thus the diffusion paths in the cathode material are shorter.

**Figure 6:**
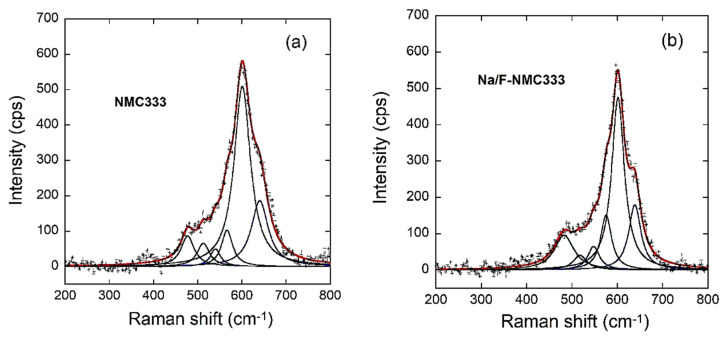
Raman scattering spectra for (**a**) NMC333 and (**b**) Na/F-NMC333 samples.

Raman spectroscopy was also applied to investigate the structural properties because this technique is sensitive to the short-range environment of oxygen coordination around the cations in oxide frameworks. The Raman active modes of the layered oxide Li*M*O_2_ (*M* = Ni, Mn, and Co) substantiate the XRD results, as they are less affected by the grain size. They correspond to vibrations that primarily involve the atomic motion of cations against their oxygen neighbors. Consequently, these modes are very sensitive to the cationic local environment in the rhombohedral crystal lattice [43]. Layered Li*M*O_2_ (*M* = Ni, Mn, or Co) compounds have the spectroscopic *D_3d_^5^* symmetry, in which the representation of the vibration modes associated to each transition-metal ion are as follows: Γ = 2*A*_2u_ + 2*E*_2u_ + *A*_1g_ + *E*_g_. Only the *A_1g_* and *E_g_* are Raman active modes, and they originate from the *M*–O stretching and O–*M*–O bending modes, respectively. In the present study, the compound has three *M* ions (*M* = Ni + Mn + Co). Therefore, as illustrated in Figure 6a,b, the three *A*_1g_ and *E*_g_ modes overlap to give two broad bands that are centered at approximately 480 and 600 cm^−1^. The best fit to the Raman spectra is achieved starting from a prescribed set of three individual bands of Lorentzian shape for the overlapping band profiles. Table 4 lists the different spectral parameters (position, width, and area) of the deconvoluted bands. They match well with those of the individual compounds: LiNiO_2_, LiCoO_2_, and LiMnO_2_ [44]. Conversely, the bandwidths of the *ν*_1_ and *ν*_2_ Ni-type vibrations for the NMC333 sample are slightly larger than those of Na/F-NMC333. A possible explanation for the broadening of the Raman bands for Ni-containing compounds is the Ni_Li_ antisite defect (Ni on the Li site). This is more evidence of the larger Ni/Li cationic disorder in this pristine sample.

**Figure 7:**
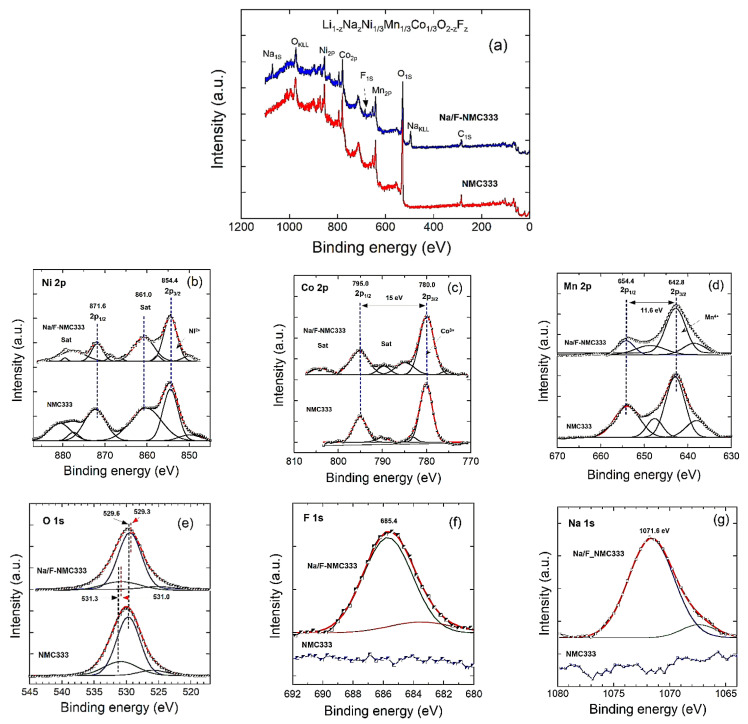
XPS results for the NMC333 and Na/F-NMC333 samples: (**a**) survey spectra and (**b**–**g**) high-resolution spectra. (**b**) Ni 2p, (**c**) Co 2p, (**d**) Mn 2p, (**e**) O1s, (**f**) F1s, and (**g**) Na1s core levels.

The chemical states of the elements in the samples were revealed through X-ray photoelectron spectroscopy. The XPS spectra of the Ni 2p, Co 2p, Mn 2p, O 1s, Na 1s, and F 1s core levels for the NMC333 and Na/F-NMC333 samples are illustrated in Figure 7. The binding energies obtained in the XPS analysis were corrected for specimen charging by referencing the C 1s line at 284.60 eV. The survey spectrum (Figure 7a) indicates the presence of Ni, Co, Mn, and O elements in both samples, with the incorporation of the Na and F cores in the Na/F-NMC333 specimen. An analysis of the Ni 2p spectrum in Figure 7b shows that the most intense Ni 2p_3/2_ peak, located at about 854.4 eV, is accompanied by a satellite at about 861.0 eV, while Ni 2p_1/2_ and its satellite are located at 871.6 and 876.7 eV, respectively. These are characteristics of the Ni^2+^ cation, and they agree well with those previously reported for Ni^2+^ [45]. The fitting of the Co 2p spectrum in Figure 7c presents two major peaks at about 780.0 and 795.0 eV, which are assigned to the Co 2p_3/2_ and Co2p_1/2_ core levels of Co^3+^, respectively, with a spin-orbit energy separation of 15.0 eV, which is in good agreement with the values reported in [46]. The weak satellite peaks at 789.5 and 804.4 eV are also the fingerprints of Co^3+^ ions in Na/F-NMC333 [47]. This implies that the co-doping does not alter the oxidation state of cobalt, but, on the contrary, stabilizes it. In the spectrum of Mn 2p (Figure 7d), two main characteristic peaks assigned to Mn 2p_3/2_ and Mn2p_1/2_ at about 642.8 and 654.4 eV, respectively, with 11.6 eV spin-orbit energy separation, indicate the majority of Mn^4+^, which is in good agreement with the values previously reported [45]. The coincident peak position of the standard Mn^4+^ reveals no side effect on the valence state of Mn after the introduction of Na and F dopants.

The XPS spectrum of O 1s in Figure 7e contains a main peak located at 529.6 eV, which is attributed to the transition-metal–oxygen (*M*-O) bonds, and a shoulder peak observed at 531.8 eV, which is assigned to the residual oxygen related to impurities with OH^−^ or O^−^ bonding on the surface [48]. The binding energies of the elements are listed in Table 5. Compared with the features of NMC333, the O 1s peaks of the Na/F-NMC333 sample are slightly shifted, which may be attributed to the fluorine ions that successfully substituted for the oxygen. In addition, the Na/F-NMC333 sample exhibits a relatively high content of the lattice oxygen, which reveals the reduction in the lithium impurities after the co-substitution of Na and F ions [31]. Furthermore, the peaks of F 1s and Na 1s can be observed in the Na/F-NMC333 sample as displayed in Figure 7f,g, respectively. The peaks at 685.4 and 1071.8 eV for the Na/F-NMC333 sample are consistent with the F^−^ and Na^+^ standard binding energies, respectively [31,49]. In particular, the measured binding energy for F 1s is 685.4 eV, which closely coincides with those of the metal fluorides NiF_2_, LiF, and MnF_2_, which lie between 684.5 and 685.9 eV (see [50] and Refs. herein). The binding energy for F 1s in the LiNi_1/3_Mn_1/3_Co_1/3_O_2_ framework is comparable to the 685 eV found for Ni-rich compounds (i.e., LiNi_0.8_Co_0.15_Al_0.05_O_2_ [51] and LiNi_0.8_Mn_0.1_Co_0.1_O_2_ [52]). Therefore, no LiF layer was formed on the surface of the particles, and this result corroborates the substitution of O^2−^ for F^−^ not only in the bulk, but also on the surface of the particles. An additional proof is given by the fact that the Mn 2p, Co 2p, and Ni 2p peaks of LiF-coated lamellar compounds show remarkable chemical shifts of the binding energy [53,54], which were not observed here. Both the pristine and co-doped samples had Co 2p, Ni 2p, and Mn 2p peaks without remarkable chemical shifts of the binding energy, which indicates that the chemical states of the TM elements in the layered LiNi_1/3_Mn_1/3_Co_1/3_O_2_ structure did not change upon doping by F^−^ and Na^+^. These results agree well with the XRD data, which confirm the substitution of Na^+^ for Li^+^, rather for TM ions, which do not change their oxidation state. This contradicts prior results on LiNi_0.8_Mn_0.1_Co_0.1_O_2_ and suggests that the Ni-rich sample is more sensitive to fluorine substitution [55]. In summary, our XPS analyses demonstrated that fluorine and sodium ions were successfully substituted for the oxygen and lithium sites, respectively.

### 2.2. Electrochemical Behavior

**Figure 8:**
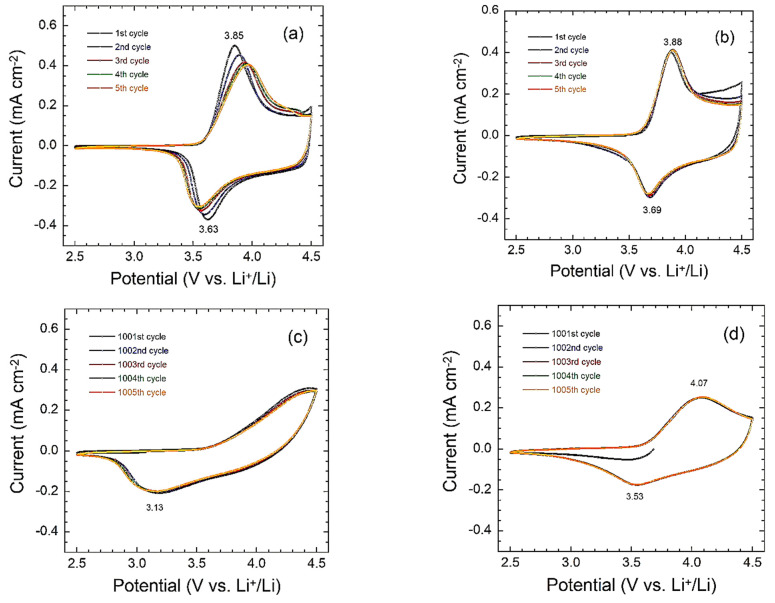
Cyclic voltammograms of lithium-ion battery cells with: (**a**,**c**) NMC333 and (**b**,**d**) Na/F-NMC333 positive electrodes in the potential range of 2.5–4.5 V vs. Li^+^/Li^0^ at a 0.02 mV s^−1^ scan rate. (**a**,**b**) Fresh cells and (**c**,**d**) cells after 1000 cycles at a 1C rate.

The as-prepared NMC333 and Na/F-NMC333 electrode materials were tested in 2032 coin-type cells using cyclic voltammetry (CV) and galvanostatic charge–discharge (GCD) experiments. The cyclic voltammograms (Figure 8) were carried out for a fresh cell and after 1000 cycles in the voltage range of 2.5–4.5 V vs. Li^+^/Li at a 0.02 mV s^−1^ scan rate. As is shown in Figure 8a,b, the fresh NMC333 and Na/F-NMC333 electrodes (first cycle) show broad anodic and cathodic peaks centered at 3.85/3.63 V and 3.88/3.69 V, respectively, which correspond to the oxidation/reduction (Ni^2+^/Ni^3+/4+^) in the layered *R*$\bar{3}$*m* lattice [56]. The redox peaks related to the possible Co^3+^/Co^4+^ oxidation/reduction did not appear in these CV curves because they are expected at a higher potential of 4.5–4.6 V vs. Li^+^/Li. Note the absence of the electrochemical activity of Mn^3+^ ions (redox peaks at ~3 V), which indicates that all the Mn ions are in the valence state +4, which is known to be electrochemically inactive in the NMC333 structure [57]. There is no significant difference in the shapes of the CV curves of both electrodes, which indicates that Na^+^/F^−^ co-doping does not alter the layered structure of NMC333 cathode materials. This result agrees well with the previous reports in [9].

The pristine sample exhibits a broader redox peak (Figure 8a) than that of the co-doped sample (Figure 8b). The co-doped sample shows sharp redox peaks with less polarization, and an initial potential difference between anodic and cathodic peaks (∆*E*_p_ = 0.19 V), which remains constant over the first five cycles. In contrast, the pristine NMC333 electrode exhibits a higher initial potential difference (∆*E*_p_ = 0.22 V), which gradually increases in the subsequent cycles. The sharpness of the redox peaks and the rather small potential difference (∆*E*_p_) in the co-doped sample, which are maintained upon cycling, indicate a better reversibility and cycling stability than the pristine one. After long-life cycling (i.e., 1000 cycles), the CV curves show significant changes: the typical redox peaks of NMC333 almost vanish, while the anodic and cathodic peaks of the co-doped electrode still display the electrochemical features of the NMC333 framework, with less sharpness and higher potential difference (∆*E*_p_ = 0.54 V). Nevertheless, this indicates an increase in the cell polarization upon cycling, accompanied by declined charge and discharge capacities.

**Figure 9:**
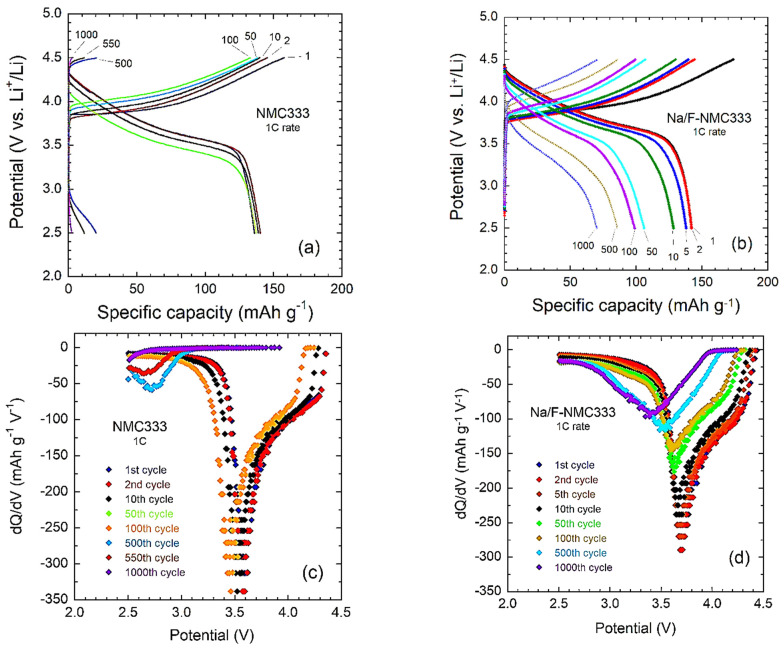
Galvanostatic charge–discharge profiles at typical cycles (1st, 2nd, 5th, 10th, 50th, 100th, 500th, and 1000th) of: (**a**) pristine NMC333 and (**b**) co-doped Na/F-NMC333, cycled at a 1C rate in the voltage range of 2.5–4.5 V. The corresponding derivative (d*Q*/d*V*) vs. voltage plots for (**c**) NMC333 and (**d**) co-doped Na/F-NMC333.

Figure 9 shows the galvanostatic charge–discharge profiles of the pristine NMC333 and co-doped Na/F-NMC333 electrodes in the voltage range of 2.5–4.5 V vs. Li^+^/Li at 0.1C and at 1C current densities. The polarization and drop in the operating voltage increase with the increasing current density. This polarization is more pronounced in the pristine electrode than in the co-doped one. At a 1C rate, we can observe charge and discharge plateaus at 3.87 and 3.70 V for the pristine and co-doped samples, respectively, which match well with the redox peak observed in their CV.

To explore the effect of Na^+^/F^−^ co-doping on the cycling stability and voltage decay, the incremental capacity (d*Q*/d*V*) was examined for both electrodes cycled at a 1C rate over 1000 cycles. The d*Q*/d*V* vs. *V* plots presented in Figure 9e,f confirm the electrochemical behaviors observed from cyclic voltammetry. After 100 cycles of charge–discharge at 1C, the evolution of the structure and the growth of a spinel-like phase are clearly depicted for the pristine NMC333 electrode, with the appearance of the broad voltage peak at ca. 2.7 V, which is the fingerprint of a thin layer of defective spinel that is consistent with the reduction in Mn^4+^ ions [58,59]. These electrode–electrolyte instabilities result in irreversible transformations at the interfaces, with the formation of an insulating layer that impedes the transport properties of the electrode, which leads to very low-capacity retention after 500 cycles. Conversely, this phase transformation is not detected for the Na/F-NMC333 electrode, in which the entire integrity is maintained over 1000 cycles. As a result, the Na/F co-doping strategy that screens the surface reactivity of the oxide preserves the charge transport and capacity retention, even at a high current density of 1C. A comparison is difficult due to the lack of electrochemical testing of LiNi_1/3_Co_1/3_Mn_1/3_O_2_ cathode materials doped with either Na or F over a great number of cycles; however, the scanty literature data are listed in Table 6. Our results for Li_0.975_Na_0.025_Ni_1/3_Mn_1/3_Co_1/3_O_1.975_F_0.025_ show better trends than those reported for the newly produced Li//Li_0.95_Na_0.05_Ni_1/3_Co_1/3_Mn_1/3_O_2_ cell, which faded quickly with about 2.51% in the first ten runs [15]. Other reports are limited at low-rate testing (<1C), and no more than 50 runs were carried out.

**Figure 10:**
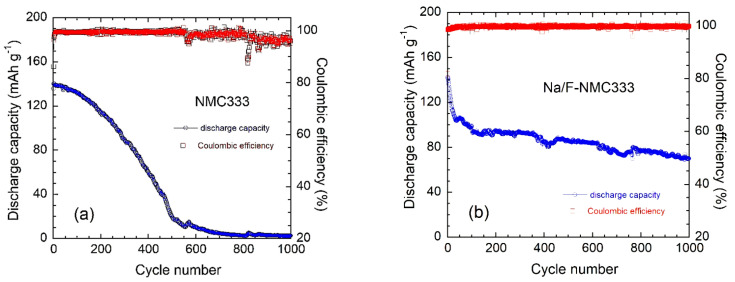
Specific discharge capacities and Coulombic efficiencies for electrodes tested at 1C over 1000 cycles in the voltage range of 2.5–4.5 V: (**a**) pristine NMC333 and (**b**) co-doped Na/F-NMC333.

The results presented in Figure 10 show the improved capacity retention for the Na/F co-doped sample compared with that of pristine NMC333. At a 1C rate, the pristine sample delivers an initial specific discharge capacity of 136 mAh g^−1^ and retains 18 mAh g^−1^ (13% capacity retention) after at 500 cycles, with a Coulombic efficiency of 99.1%. The Na/F-NMC333 electrode delivers a specific capacity of 142 mAh g^−1^ and retains 86 mAh g^−1^ (60% capacity retention) after 500 cycles, with a Coulombic efficiency of 99.8%. After the long-life test (i.e., 1000 cycles at 1C rate), the specific discharge capacity for the co-doped sample was still 70 mAh g^−1^, which corresponds to a capacity retention of ~50%, while the pristine electrode no longer worked. The Na/F-NMC333 electrode shows remarkable low-capacity fading, with a rate of 0.05% per cycle over 1000 charge–discharge runs. In contrast, the pristine NMC333 electrode delivers a residual capacity of 2.5 mAh g^−1^. The results are summarized in Table 7.

**Figure 11:**
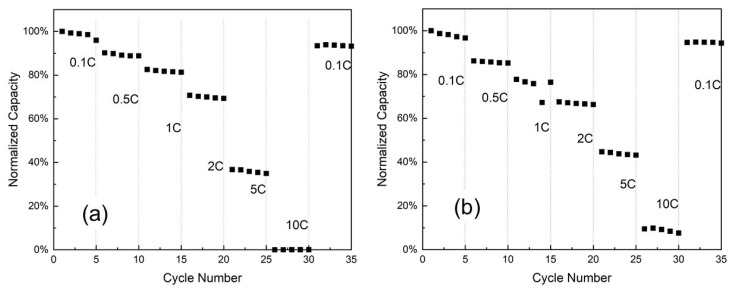
Rate capabilities for: (**a**) pristine NMC333 and (**b**) co-doped Na/F-NMC333 at various C-rates in the range of 0.1C–10C. For both electrodes, the capacity is normalized relative to the first discharge capacity at a 0.1C rate.

Figure 11 presents the rate capability of the NMC333 and Na/F-NMC333 electrodes cycled in the voltage range of 2.5–4.5 V at various C-rates (from 0.1C to 10C). For both electrodes, the capacity is normalized relative to the first discharge capacity at a 0.1C rate. The discharge capacity decreases moderately upon increasing C-rates for both samples, which exhibit almost the same initial rate capability when cycled from 0.1 to 2C. In contrast, at 5C and 10C, the co-doped sample shows better capacity retention. This improvement in the electrochemical performance illustrates the importance of Na^+^ doping for the layered LiNi_1/3_Co_1/3_Mn_1/3_O_2_ that extends the interslab spacing of Li-O and, hence, facilitates fast Li diffusion. As a consequence, the extraction/insertion mechanism (charge–discharge process) of lithium ions is highly reversible into a Na/F co-doped layered structure. In addition, this doping decreases the degree of cation mixing (Li^+^/Ni^2+^), which retards the electrode polarization and enhances the structural stability [17]. This improved electrochemical performance is mainly attributed to the synergistic effect of Na^+^ and F^−^ doping [60].

**Figure 12:**
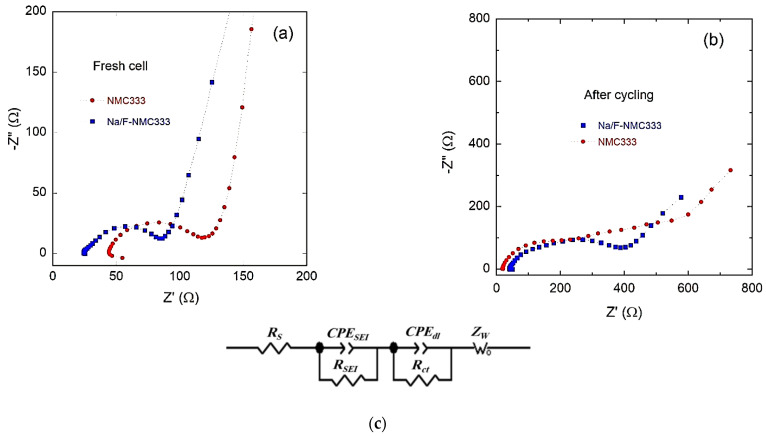
EIS measurements of NMC333 and Na/F-NMC333 electrodes. Nyquist plot of: (**a**) fresh electrodes and (**b**) electrodes after cycling at 1C rate. (**c**) Equivalent circuit used for the analysis of the -Z” vs. Z’ planes.

To further shed light on the electrochemical properties of NMC333 and Na/F-NMC333 electrode materials, EIS measurements were carried out before cycling (fresh cells) and after 1000 cycles at 1C rate, as shown in Figure 12a,b. The equivalent circuit model used to analyze the Nyquist plots is shown in Figure 12c. The Nyquist plots shown in Figure 12a,b can be divided into four components: (i) the intercept at high frequency with the Z’-axis, which is related to the uncompensated ohmic resistance of the cell (*R*_s_); (ii) the first depressed semicircle in the high-frequency region, which is associated with the impedance of the SEI layer (*R*_SEI_, CPE_SEI_); (iii) the depressed semicircle in the medium-frequency region, which is ascribed to the charge-transfer impedance and interfacial capacitance at the electrode/electrolyte interface (*R*_ct_, CPE_dl_); (iv) the inclined line in the low-frequency range, which is due to the Li^+^-ion diffusion-controlled process characterized by the Warburg impedance: Z_W_(ω) = σ_w_ (1 − j) ω^−1/2^, where σ_w_ is the Warburg factor, ω is the frequency, and j = $\sqrt{-1}$ [61]. From the fitted parameters listed in Table 8, it can be seen that the general trend is an increase in the total impedance after 1000 cycles at a 1C rate for both electrodes. The relatively high value of the initial internal resistance (*R*_s_) is attributed to the freshly assembled cell with uncycled electrodes. The *R*_SEI_ and *R*_ct_ show significant increases upon cycling, which provoke, as is shown in the GCD curves, an increase in the cell polarization after long-life cycling. The real part (*Z*’(ω)) of the total impedance of the cell is the sum of the real parts of the four components:

*Z*’(ω) = *R*_s_ + *R*_SEI_ + *R*_ct_ +*Z*_w-R_. (3)

*Z*_w-R_ is obtained from the fit of the Nyquist plots (Table 8), and the apparent diffusion coefficient (*D*_Li_) is given according to the relation [62]:

(4) $$D_{\mathrm{Li}}=\frac{R^{2}T^{2}}{2A^{2}n^{4}F^{4}C_{\mathrm{Li}}{}^{2}Z_{w-R}{}^{2}}$$

in which *R* is the gas constant, *T* is the absolute temperature, *F* is the Faraday’s constant, *n* is the number of electrons transferred, *C*_Li_ is the concentration of Li^+^ ions inside the electrode, and *A* is the effective surface area of the electrode.

The values of the apparent diffusion coefficient (*D*_Li_) in the NMC electrodes before and after cycling are listed in Table 8. For a fresh Na/F-NMC333 electrode, the *D*_Li_ is 1.7 × 10^−11^ cm^2^ s^−1^, which is higher than that for the NMC333 electrode (1.3 × 10^−11^ cm^2^ s^−1^). These *D*_Li_ values match well with those of the literature and are within the range of from 10^−11^ to 10^−14^ cm^2^ s^−1^ [5,63]. A comparison of the kinetics confirms that the co-doping by Na and F facilitates the Li-ion diffusion due to several factors, such as the larger Li-layer spacing, the lower Li/Ni cation mixing, and the increased structural stability. After long-life cycling (1000 cycles at a 1C rate), the diffusion coefficients of Li^+^ ions for both electrodes decrease from ~10^−11^ to ~10^−14^ cm^2^ s^−1^, which is due to the electrode aging.

More information on the change in the overall cell potential as a function of the depth of charge (DOD) can be obtained by evaluating the area-specific impedance (ASI) (expressed in Ω cm^2^), which is given by the following relation [64]:

(5) $$ASI=A\frac{OCV-V_{cell}}{I},$$

where *A* is the cross-sectional area of the electrode, Δ*V* = *OCV*-*V*_cell_ is the potential change during current interruption for 60 s at each DOD, and *I* is the current passed throughout the cell. Various factors can affect the area-specific impedance, including ohmic drop, Li-ion transport through the electrolyte, and solid-state diffusion within the electrode. Moreover, ASI does not need to reach equilibrium conditions, as is the case with EIS, which makes this technique more representative for the total-internal-resistance evaluation during cycling.

**Figure 13:**
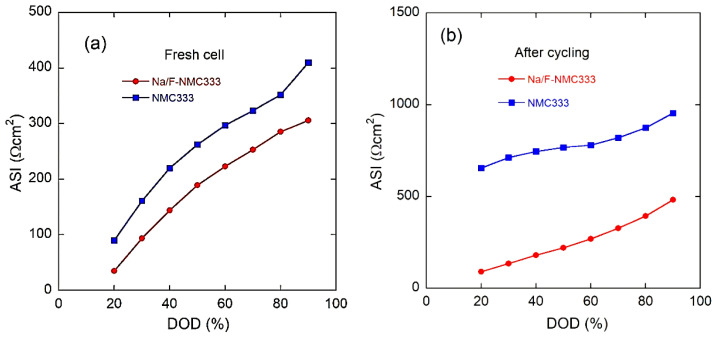
Area-specific impedances of NMC333 and Na/F-NMC333 as a function of depth of discharge (DOD): (**a**) for a fresh cell and (**b**) after 1000 cycles at a 1C rate.

Figure 13a,b displays the variation in the ASI for the NMC333 and Na/F-NMC333 electrodes before and after 1000 cycles at a 1C rate, respectively. At 90% DOD, the ASI values at the first cycle are 360 and 305 Ω cm^2^, and they increase to 696 and 482 Ω cm^2^ after long-life cycling (1000 cycles) for NMC333 and Na/F-NMC333, respectively. These results indicate that the charge-transfer resistance is dependent on the DOD, as well as the aging of the electrode. In addition, at the 1000th cycle, the rate of increase in the ASI values for the pristine NMC333 is much higher than that observed for the doped Na/F-NMC333. As is seen in Figure 13b, the ASI value at 20% DOD for the fresh NMC333 electrode is about 56 Ω, which increased to 409 Ω after 1000 cycles. This increase is smaller in the case of Na/F-NMC333, as the ASI value increased only slightly from 35 to 90 Ω. This confirms that Na/F doping may not only inhibit the rock-salt formation on the surface to form a more stable SEI film during cycles [65], but also cause a decrease in the charge-transfer resistance and facilitate ion and electron transfer during the charge–discharge process. This reduction in the ASI values, and, hence, increase in the Li-ion diffusion, may be attributed to the observed larger interslab spacing distance (i.e., LiO_2_ (*I*_LiO2_)), which favors Li-ion diffusion. These results agree well with those reported by Belharouak et al. [64] for the LiNi_1/3_Co_1/3_Mn_1/3_O_2_ electrode, and by Oh et al. [65] for Li[Ni_0.5_Mn_0.5_]_1-x_Co_x_O_2_.

## 3. Conclusions

In this study, we successfully applied Na^+^ and F^−^ co-doping to improve the structural stability and electrochemical performance of LiNi_1/3_Co_1/3_Mn_1/3_O_2_ cathodes. The pristine and co-doped materials were synthesized via the sol–gel method by using EDTA as a chelating agent, and were characterized by XRD, SEM, TEM, EDS, Raman, and XPS. The EDS and XPS results confirm a successful Na^+^ substitution for Li^+^ and F^−^ substitution for oxygen in the Na/F-NMC333 sample. Structural studies revealed that the Na^+^ and F^−^ co-doping has several effects: (i) both the *a*- and *c*-axis parameters are slightly enlarged (i.e., 0.2 and 0.1%, respectively); (ii) the incorporation of Na into the Li site reduces the Ni/Li cation mixing from 2.08% for pristine material to 1.28% for Na/F-NMC333; (iii) Na insertion enlarges the Li-slab space by 7.2%, which favors Li-ion transport. The Raman spectroscopy provided additional evidence that the Na/F-NMC333 sample has less cation mixing than the pristine sample. The SEM and TEM results show that there is no significant difference in the particle size (i.e., an average particle size of ~190 nm, and a porous framework with an average pore size of 3 nm). Electrochemical characterizations show that the Na/F-NMC333 sample has improved electrochemical properties. The improved rate capability is attributed to the enhanced Li-ion conductivity, determined by EIS and ASI experiments, which is due to the enlarged Li slab. A comparison of the kinetics confirms that the co-doping by Na and F facilitates the Li-ion diffusion due to several factors, such as the larger Li-layer spacing, the lower Li/Ni cation mixing, and the increased structural stability. After long-life cycling (1000 cycles at 1C rate), the diffusion coefficients of Li^+^ ions for both electrodes decrease from ~10^−11^ to ~10^−14^ cm^2^ s^−1^, which is due to the electrode aging. The improved cycle ability is attributed to the increased structural stability, which is due to the lower cation mixing and the stabilization of the surface structure. Consequently, the Na/F-NMC333 electrode demonstrated an outstanding cycling ability, with a capacity fading of 0.05% per cycle over 1000 cycles at a 1C rate.

## 4. Materials and Methods

Pristine LiNi_1/3_Mn_1/3_Co_1/3_O_2_ (NMC333) and Na/F-co-doped Li_0.975_Na_0.025_Ni_1/3_Mn_1/3_Co_1/3_O_1.97_F_0.025_ (Na/F-NMC333) samples were synthesized by the sol–gel method using EDTA as a chelating agent. Stoichiometric amounts of CH_3_COOLi·2H_2_O, Ni(CH_3_COO)_2_·4H_2_O, Co(CH_3_COO)_2_·4H_2_O, and Mn(CH_3_COO)_2_·4H_2_O (analytical grade, 99.99%, Sigma-Aldrich) were used as starting materials for the pristine sample. NaF (Analytical Rasayan) was added for preparing the co-doped sample. The stoichiometric amounts were mixed together and were then dissolved in deionized water by stirring for 1 h. The molar ratio of the chelating agent (EDTA) to the total metal ions was adjusted to unity and then added step-by-step wisely into the stirred aqueous solution of all the metal cations at a well-defined pH concentration and temperature. The pH of the solution was adjusted at ~7 using an alkaline solution of ammonium hydroxide, and the temperature was set at 80 °C. The prepared solution was stirred using magnetic stirring to evaporate until a transparent sol–gel was obtained. Next, the obtained gel was dried in vacuum. The resulting precursors were heated at 450 °C for 5 h, and then ground and recalcined at 750 °C for 10 h with intermittent grinding.

The crystal structure of the final product was determined by XRD using a Philips X’Pert apparatus equipped with a CuK_α_ X-ray source (λ = 1.54056 Å) in the 2*θ* range of 10–80°. The morphology of the materials was studied by field emission scanning electron microscopy (FESEM) (JSM-7800F microscope, JEOL, Tokyo, Japan) and transmission electron microscopy (TEM) (JEM-2100 microscope, JEOL, Tokyo, Japan). The BET surface area and pore size distribution of the synthesized samples were determined from N_2_-adsorption experiments (ASAP 2020, Micromeritics, Norcross, GA, USA). X-ray photoelectron spectra were recorded using a scanning X-ray microprobe system (PHI VersaProbe II, Ulvac-Phi, Chanhassen, MN, USA) equipped with a Mg K_α_ source (λ = 1253.6 eV). Raman spectra were recorded at room temperature with a micro-Raman spectrometer (Renishaw, Wotton-under-Edge, UK), equipped with a confocal Raman microscope inViaTM system at a 532 nm laser-line excitation. The spectra were calibrated with the reference Si phonon peak at 520 cm^−1^.

The positive electrodes of lithium cells were fabricated as a mixture of active materials (NMC), carbon black (CB), and polyvinylidene fluoride binder (PVDF) in an 8:1:1 mass ratio. The mixture was added to *N*-methyl-2-pyrrolidone (NMP) solvent. The mixed slurry was magnetically stirred for 24 h to form a homogeneous blend. The well-blended slurry was cast on an aluminum foil by a doctor blade, and dried under vacuum at 100 °C for 24 h. Finally, the electrodes were punched out as ~0.97 cm^2^ discs (Ø = 11 mm). CR2032 coin cells were processed in an argon-filled glovebox using 30 µL electrolyte dripped on the electrode, and then on a Celgard 2400 separator. Electrochemical tests were carried out using an Arbin BT2000 battery cycler at room temperature. Before cycling, cells were initially maintained at rest for 30 min. Cells were cycled galvanostatically at C/10 and 1C rates, in the voltage range of 2.5–4.5 V vs. Li^+^/Li. Cyclic voltammetry was conducted at room temperature on a BioLogic VSP workstation (Biologic Sci. Instr., Knoxville, TN, USA), in which the potential was set to sweep from open-circuit voltage to 4.5 V, and then to sweep back to 2.5 V at a 0.02 mV s^−1^ scanning rate. Electrochemical impedance spectroscopy was also conducted by the VSP workstation, in the frequency range of from 500 kHz to 0.1 Hz, with an amplitude of 5 mV.

## Figures and Tables

**Table 1:** Structural parameters obtained from Rietveld refinements of X-ray diffractograms of NMC333 and Na/F-NMC333 samples.

Crystal Data	NMC333	Na/F-NMC333
Lattice parameters		
*a (*Å*)*	2.857(3)	2.859(7)
*c(*Å*)*	14.205(3)	14.221(6)
*V (*Å^3^*)*	100.43	100.72
*c/a*	4.971(9)	4.974(1)
*L_c_* (nm)	42	34
*ε* × 10^−2^ (rd)	1.84	1.16
*I_(_*_003)_/*I*_(104)_	1.24	1.32
Reliability factors		
*R_p_ (%)*	9.8	7.8
*R_wp_ (%)*	11.9	12.2
*χ^2^*	1.22	1.61
Occupancy (Occ)		
Ni^2+^ on Li-site%	2.08	1.28
Na on Li-site%	0	2.47
F on O-site%	0	2.39
*Z_oxy_*	0.24056	0.24499
*S*_(MO2)_ (Å) ^a^	2.636	2.513
*I*_(LiO2)_ (Å) ^b^	2.099	2.251

^a^*S*_(MO2)_*= 2((1/3)−Z_oxy_)c* is the thickness of the metal–O_2_ planes. ^b^ *I*_(LiO2)_ *= c/3−S_(MO2)_* is the thickness of the interslab space.

**Table 2:** SEM–EDS analyses of NMC333 and Na/F co-doped NMC333 samples.

Nominal Formula	Mole Fraction					
	Na	F	Ni	Mn	Co	O
LiNi_0.33_Mn_0.33_Co_0.33_ O_2_	-	-	0.3411	0.3305	0.3284	1.998
Li_0.975_Na_0.025_Ni_0.33_Mn_0.33_Co_0.33_O_1.975_F_0.025_	0.028	0.026	0.3240	0.3310	0.3454	1.972

**Table 3:** BET parameters of the NMC333 and Na/F-NMC333 samples.

Material	Specific Surface Area (m^2^ g^−1^)	Average Pore Radius (nm)	Pore Volume (cm^3^ g^−1^)	*L*_BET_ (nm)
NMC333	6.7	2.8	0.0075	196
Na/F-NMC333	7.1	3.0	0.0086	185

**Table 4:** Analysis of the Raman active modes (*E*_g_ and *A*_1g_) for (a) NMC333 and (b) Na/F-NMC333 samples using Lorentzian profiles. Band positions are given with an accuracy of ±1 cm^−1^.

Modes		Band Position (cm^−1^)	Band Width (cm^−1^)	Band Area
NMC333				
ν1	Eg (Ni)	477	52	5678.4
ν2	A1g Ni)	539	31	3226.7
ν3	Eg (Co)	512	37	2371.7
ν4	A1g (Co)	600	45	25,298.9
ν5	Eg (Mn)	566	41	5950.3
ν6	A1g (Mn)	640	51	9213.5
Na/F-NMC333				
ν1	Eg (Ni)	480	45	6151.7
ν2	A1g (Ni)	544	27	4840.6
ν3	Eg (Co)	514	34	3556.9
ν4	A1g (Co)	601	44	23,604.8
ν5	Eg (Mn)	571	39	6241.5
ν6	A1g (Mn)	638	49	12,607.1

**Table 5:** Results of the XPS analysis for NMC333 and Na/F-NMC333 samples: binding energy (eV) of Ni, Co, Mn, O, F, and Na elements. ΔE is the binding energy (eV) separation. Band positions are given with an accuracy of ±0.1 eV.

Element	Core Level	Binding Energy (eV)	
		NMC333	Na/F-NMC333
Ni 2p	2p_3/2_	845.4	845.4
	2p_1/2_	871.8	871.6
	Satellite	861.6	876.7
	ΔBE	17.4	17.2
Co 2p	2p_3/2_	780.0	780.0
	2p_1/2_	795.0	795.0
	Satellite	789.4	790.0
	ΔBE	15.0	15.0
Mn 2p	2p_3/2_	742.8	742.8
	2p_1/2_	754.4	754.4
	ΔBE	11.6	11.6
O 1s	1s	529.6	529.3
		531.3	531.0
F 1s	1s	-	685.4
Na 1s	1s	-	1071.8

**Table 6:** Comparison of the cyclability of LiNi_1/3_Co_1/3_Mn_1/3_O_2_ cathode materials doped with either Na or F elements. Specific capacity values are obtained after cycling.

Dopant	Specific Capacity (mAh g^−1^)	Cycles	Decay per Cycle (%)	Ref.
0.05 Na	120 @ 1C	10	2.51	[15]
0.03 Na	133 @ 0.5C	50	0.09	[16]
0.05 Na	177 @ 0.1C	30	0.42	[17]
0.1 Na	95 @ 0.2C	100	0.37	[18]
0.08 F	136 @ 1.2 mA cm^−2^	50	0.21	[20]
0.04 F	172 @ 0.2C	30	0.15	[21]
0.04 Al + 0.05 F	148 @ 0.1C	20	0.3	[22]
0.02 Al + 0.02 B + 0.02 F	157 @ 1C	30	0.02	[23]
0.025 Na + 0.025 F	70 @ 1C	1000	0.05	this work

**Table 7:** Electrochemical performance of pristine NMC333 and Na/F-NMC333 electrodes of coin-type cells tested at a 1C rate in the voltage range of 2.5–4.5 V vs. Li^+^/Li. Capacity decay (in mAh g^−1^) per cycle is calculated for 500 cycles.

Electrode	Specific Capacity (mAh g^−1^)				Coulombic Efficiency 200th Cycle (%)
	1st Cycle	500th Cycle	Capacity Decay	1000th Cycle	
NMC333	136	17	0.24	2.5	99.4
Na/F-NMC333	142	86	0.11	70	99.5

**Table 8:** Fitting results of Nyquist plots of NMC333 and Na/F-NMC333 electrodes before and after 1000 cycles at a 1C rate.

Parameters	NMC333		Na/F-NMC333	
	Fresh Cell	After Cycling	Fresh Cell	After Cycling
*R*_s_ (Ω)	45.4	18.1	29.4	51.7
*R*_SEI_ (Ω)	-	164.7	-	95
CPE_SEI-T_	-	1.2 × 10^−5^	-	1.4 × 10^−5^
CPE_SEI-P_	-	0.82	-	0.81
*R*_ct_ (Ω)	61.6	321.3	62.8	242.5
CPE_dl-T_	1.5 × 10^−5^	3 × 10^−4^	6.8 × 10^−5^	8.9 × 10^−5^
CPE_dl-P_	0.85	0.65	0.74	0.73
Z_w-R_	51.1	2073	46	950.6
Z_w-T_	0.097	31.6	0.123	12.8
Z_w-P_	0.58	0.56	0.78	0.57
*D*_Li_^+^ (cm^2^ s^−1^)	1.3 × 10^−11^	8.3 × 10^−15^	1.7 × 10^−11^	4.0 × 10^−14^

## Data Availability

All data are included in this article.
